# Maternal pre-pregnancy overweight/obesity and gestational diabetes interaction on delayed breastfeeding initiation

**DOI:** 10.1371/journal.pone.0194879

**Published:** 2018-06-18

**Authors:** Tanara Vogel Pinheiro, Marcelo Zubaran Goldani, IVAPSA group

**Affiliations:** 1 Department of pediatrics, Universidade Federal do Rio Grande do Sul, Porto Alegre, Rio Grande do Sul, Brazil; 2 Department of pediatrics, Hospital de Clínicas de Porto Alegre, Porto Alegre, Rio Grande do Sul, Brazil; University of North Carolina at Chapel Hill, UNITED STATES

## Abstract

**Background:**

Cumulative evidence indicates an association between maternal overweight and gestational diabetes with delayed breastfeeding initiation; however, the presence of both conditions simultaneously has been little explored. This study aims to investigate the interaction between maternal overweight/obesity and gestational diabetes on breastfeeding initiation.

**Methods:**

This study comprises data from the IVAPSA Birth Cohort, a prospective follow-up of mothers and their newborns. Two of the five groups from IVAPSA were evaluated, considering women with and without gestational diabetes. These women were further categorized according to their pre-pregnancy body mass index as normal weight or overweight/obese.

**Results:**

219 women were evaluated, 53.4% of them had pre-pregnancy overweight/obesity and 32% had gestational diabetes. Most women were able to initiate breastfeeding within 12 hours from delivery (92.7%) and only eight (3.7%) women had not breastfed in the first 24 hours postpartum. Of these, seven were overweight/obese (77.8%) and five had gestational diabetes (66.7%), with four of them having overweight/obesity and gestational diabetes concomitantly. Women with both adverse conditions had an adjusted relative risk of delayed breastfeeding initiation of 1.072 (95% CI 1.006; 1.141), *p* = 0.032.

**Conclusions:**

The results indicate an additive interaction between maternal pre-pregnancy overweight/obesity and gestational diabetes on delayed breastfeeding initiation.

## Introduction

The prevalence of overweight and obesity among women of reproductive age has risen all over the world during the last decades [1, 2] and has been associated with a greater risk of pregnancy, obstetric and neonatal complications [3], including greater difficulties to initiate breastfeeding (BF) after delivery compared with healthy weight women [4–6]

The increase of maternal body mass index (BMI) is strongly associated with a higher risk of developing gestational diabetes (GDM) during pregnancy [7–9], which in turn is also a risk factor for BF failure [10, 11]. GDM has been associated with delayed onset of lactogenesis II (onset of copious milk secretion in the first few days postpartum), lower rates of BF initiation and earlier weaning [12].

The health benefits of BF are of particular importance for mothers with overweight and/or gestational diabetes, since BF is associated with a decreased probability of type 2 diabetes [13] and a potential decrease of postpartum weight retention [14, 15]. Children born from those women are also benefited by BF since increasing evidence indicates a protective effect against obesity and diabetes for children as well [16–18].

Considering that women with overweight and gestational diabetes are at increased risk of failure to initiate BF and that the timing of BF initiation is critical to BF success [19, 20], this study aims to investigate the interaction between maternal overweight/obesity and gestational diabetes on the timing of BF initiation.

## Materials and methods

### Participants

The subjects in this study are part of the “Impact of Perinatal Different Intrauterine Environments on Child Growth and Development in the First Six Months of Life—IVAPSA study”, a prospective follow-up of mothers and their newborns that aims to investigate health outcomes after exposure to adverse intrauterine environments. The IVAPSA study has a convenience sample of 400 mother-offspring pairs, recruited in three public hospitals of Porto Alegre, Brazil. The hospitals are Baby-Friendly, meaning that they follow the Ten Steps to Successful Breastfeeding and have written policies for breastfeeding protection, promotion and support. A detailed description of the IVAPSA recruitment and interviews has been previously published elsewhere [21].

This paper comprises only two of the five groups that integrate the IVAPSA study: women with gestational diabetes (GDM) and their offspring and a control group of mother-offspring with none of the adverse conditions investigated by IVAPSA. Women with positive test for HIV, multiple pregnancy or preterm delivery (<37 weeks) and infants with acute diseases or congenital birth defects were not included in the study. All women and babies were together in the maternity ward of the hospitals in the moment of recruitment, meaning that mother and child were healthy and with no greater health risks.

### Data collection

Information presented in this paper was collected during two interviews: the first conducted between 24 and 48 hours postpartum, in the maternity ward of the hospitals, and the second between 30 and 45 days after delivery, either in the participants’ house or in clinics located in one of the hospitals.

In addition to the information provided by the mothers during the interviews, we also collected data from the mother’s prenatal care booklet, which contains medical records of the entire gestation period, and from the hospital’s medical registries for detailed information on hospital admission, delivery, exams and hospital discharge.

### Maternal pre-pregnancy body mass index

Maternal pre-pregnancy weight was collected from the prenatal care booklet or, in the absence of this information, was reported by the mother in the first interview in response to the question “Just before you got pregnant with your new baby, how much did you weight?”. Maternal height was measured in three occasions: before delivery (by hospital staff), at 6–8 days and at 30–45 days after delivery (by trained researchers). We preferably used the last measure (30 days) because there may be a postural adjustment during pregnancy and in the first days after delivery [22]. Pre-pregnancy body mass index (BMI) was calculated by the equation “Pre-pregnancy weight (kg)/height squared (m^2^)” and categorized as Normal weight: 18.5–24.9 kg/m^2^; Overweight: 25.0–29.9 kg/m^2^; or Obese: ≥30.0 kg/m^2^. Women under 20 years were evaluated using the WHO AnthroPlus software BMI-for-age Z-score. We applied the BMI-for-age cut-offs suggested by WHO: Normal weight: Z-score between -2SD and +1SD; Overweight: Z-score >+1SD; and Obese: Z-score >+2SD.

### Gestational diabetes

The Brazilian Society of Diabetes defines gestational diabetes as any level of glucose intolerance with onset or diagnosis during gestation [23]. Threshold values for diagnosis of GDM recommended by the Brazilian Society of Diabetes are: fasting plasma glucose ≥ 92 mg/dL, 1-h plasma glucose after a 75 g oral glucose load ≥ 180 mg/dL or 2-h plasma glucose after a 75 g oral glucose load ≥ 153 mg/dL. The diagnosis of gestational diabetes was identified by review of hospital medical records and later confirmed through detailed questions about diabetes during the in-hospital interview with the mother.

### Breastfeeding initiation

During the first interview, conducted between 24 and 48 hours postpartum, the mothers were asked if they had breastfed their child any time until that moment. If the answer was positive, they were asked to indicate how long (in minutes or hours) after delivery they had breastfed for the first time. The answer was categorized in more or less than 24 hours after delivery and it is presented as the primary outcome in the analysis.

### Ethics

Approval to conduct this study has been granted by the Human Research Ethics Committees of the Hospital de Clínicas de Porto Alegre (reference n° 110097) and of the Grupo Hospitalar Conceição (reference n° 11027).

### Statistical analysis

Characteristics of the study sample according to pre-pregnancy BMI and gestational diabetes were compared using Pearson’s Chi Square test, Student T-test, and Fischer’s Exact test, as appropriate. The frequency of BF initiation in the first 24 hours after delivery was compared according to pre-pregnancy BMI and gestational diabetes diagnosis using Pearson’s Chi Square test with Bonferroni correction. Poisson regression with robust error variance was used to estimate the adjusted relative risk of failure to initiate BF in the first 24 hours after delivery, with normal weight women without gestational diabetes as the reference group. All statistical tests were two-sided and p<0.05 was considered statistically significant. Underweight women (n = 5) and women with type 1 diabetes (n = 2) and type 2 diabetes (n = 2) were not included in the analyses due to their reduced prevalence in the sample. All statistical analyses were performed using SPSS software (version 20.0; SPSS Inc, Chicago, IL).

## Results

**Fig 1:**
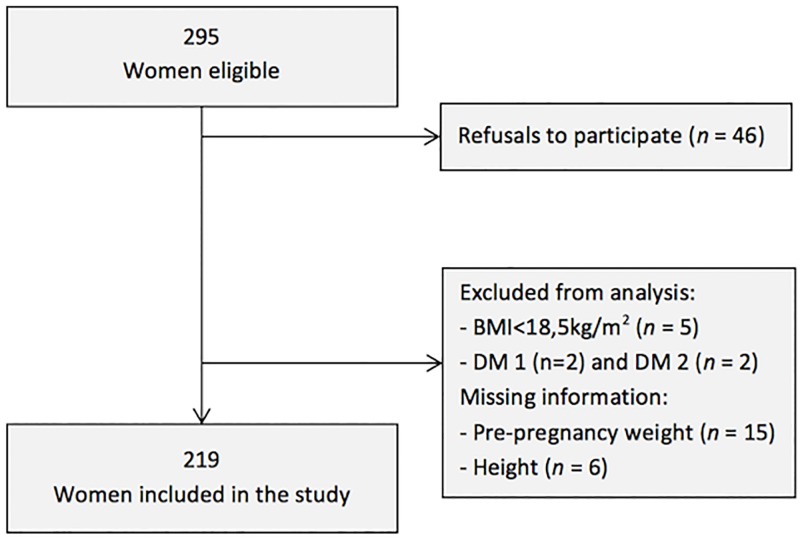
Progress of participants through the study.

Within 295 eligible women, 46 refused to participate (15.6%). Underweight women and women with type 1 or 2 diabetes were not included in the analyses due to their reduced frequency in the sample. Complete information on maternal BMI and BF initiation were available for 219 participants (Fig 1).

Women in the included sample had no statistically significant differences compared to those who refused to participate regarding race/ethnicity (p = 0.856), schooling (p = 0.628), parity (p = 0.718) and type of delivery (p = 0.476), however the participants were significantly younger than those who refused to participate [mean (SD): 26.75 (6.8) *vs*. 29.10 (7.0) years, *p* = 0.031)].

Characteristics of the study sample according to pre-pregnancy BMI and gestational diabetes (GDM) are described in Table 1. Pre-pregnancy BMI was higher in those with GDM compared to those without GDM in both categories of BMI. The frequency of multiparous women among overweight/obese was greater than among their healthy weight counterparts. Additionally, having GDM raised the frequency of cesarean delivery, but only among overweight/obese women.

**Table 1:** Characteristics of the study sample according to pre-pregnancy body mass index and gestational diabetes, collected at 24–48 hours after delivery.

	Normal Weight (*n* = 102)			Overweight/Obese (*n* = 117)			*P*^3^
	No GDM (*n* = 85)	DGM (*n* = 17)	*P*^1^	No GDM (*n* = 64)	DGM (*n* = 53)	*P*^2^	
Age (years)^a^	25.3 (7.3)	28.4 (5.8)	0.109	27.0 (6.5)	28.1 (6.2)	0.360	0.083
Pre-pregnancy BMI (kg/m^2^)^a^	21.7 (1.8)	22.7 (1.7)	**0.026**	29.4 (3.9)	31.2 (5.5)	**0.036**	**<0.001**
Schooling^b^			1.000			0.096	0.268
< 8 years	14 (16.5)	2 (11.8)		10 (15.6)	15 (28.3)		
≥ 8 years	71 (83.5)	15 (88.2)		54 (84.4)	38 (71.7)		
Parity^b^			0.425			0.222	**0.014**
None	44 (51.8)	7 (41.2)		25 (39.1)	15 (28.3)		
≥1	41 (48.2)	10 (58.8)		39 (60.9)	38 (71.7)		
Type of delivery^b^			0.260			**0.021**	0.623
Vaginal	57 (67.1)	14 (82.4)		48 (75.0)	29 (54.7)		
Cesarean	28 (32.9)	3 (17.6)		16 (25.0)	24 (45.3)		

Chi Square and Fisher’s exact test used to compare categorical variables; t test used to compare continuous variables.

^a^ continuous variables, presented as mean (SD);

^b^ categoric variables, presented as *n* (%)

^1^ *P*-value for comparison of women with and without GDM among the Normal weight group;

^2^ *P*-value for comparison of women with and without GDM among the Overweight/obese group;

^3^ *P*-value for comparison of women Normal weight and women Overweight/obese.

Most women were able to initiate BF within 12 hours from delivery (92.7%). Only eight (3.7%) women did not breastfeed in the first 24 hours postpartum. Of these, seven were overweight/obese (77.8%) and five had gestational diabetes (66.7%), with four of them having both conditions.

**Fig 2:**
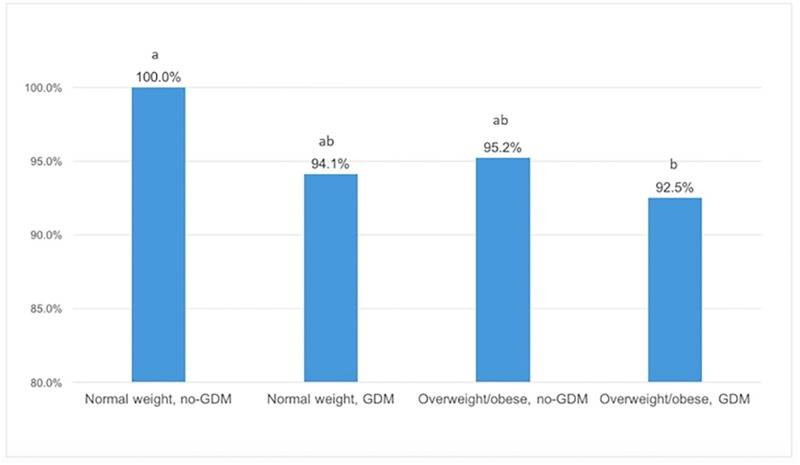
Percentage of mothers that initiated BF in the first 24 hours after delivery, according to pre-pregnancy body mass index and gestational diabetes. Chi Square test with Bonferroni correction. Distinct superscript letters represent statistical difference (p<0.05).

The percentage of mothers that did not breastfeed in the first day postpartum, according to pre-pregnancy BMI and GDM is presented in Fig 2. All women with a healthy pre-pregnancy weight without GDM managed to breastfeed in the first 24 hours postpartum. Among women with GDM and healthy weight 5.9% did not breastfeed in this period, while among overweight/obese women without GDM 4.8% had delayed initiation of BF. When both conditions were present, the frequency of delayed initiation raised to 7.5% (*p* = 0.021).

Table 2 indicates Poisson Regression analysis for the risk of not BF during the first 24h after delivery according to pre-pregnancy BMI and gestational diabetes, adjusted for cesarean delivery. Women who had both overweight/obesity and gestational diabetes presented the highest risk for delayed BF initiation among all groups.

**Table 2:** Adjusted relative risk for failure to initiate BF during the first 24 hours after delivery.

	aRR (95% confidence interval)	*p*
Normal weight, no-GDM	1 (reference)	-
Normal weight, GDM	1.063 (0.956; 1.182)	0.257
Overweight/Obese, no-GDM	1.050 (0.998; 1.105)	0.060
Overweight/Obese, GDM	1.072 (1.006; 1.141)	0.032

aRR, Adjusted Relative Risk; GDM, Gestational Diabetes. Poisson regression with robust error variance, adjusted for type of delivery

## Discussion

The results of this study indicate an additive interaction between maternal pre-pregnancy overweight/obesity and gestational diabetes on delayed BF initiation after 24 hours postpartum. This result added a new insight to other studies that also found worse outcomes for BF initiation in overweight and obese women [4, 5, 17, 24] and in women with gestational diabetes [10, 11, 25].

There is an international consensus on the importance of early BF initiation, since it has been strongly associated with reduced neonatal and early infant mortality [26–28]. Smith et al., conducted a large systematic review and meta-analysis and found that mortality risk for infants who were breastfed for the first time after 24 hours was more than 100% higher compared with infants that were breastfed within the first hour of life [28]. Also, previous studies indicate that women who did not breastfeed in the first days after delivery were less able to sustain any or exclusive BF 4 weeks postpartum [19], and had earlier weaning [29].

Poor lactation performance in overweight and obese women may be explained by several different aspects, such as: abnormal development of mammary glands before, during and after pregnancy [30, 31]; complications of pregnancy, including higher rates of cesarean section [9, 32], preterm birth [9, 32], and babies with macrosomia [9, 33]; psychosocial, economic and cultural factors, such as postpartum depression [34, 35], body image concerns [36, 37], education and income [38]; maternal confidence and behavior beliefs [38]; latching and positioning difficulties [39–41]; less support from family, friends and hospital staff [38, 42, 43]; and hormonal and metabolic alterations [44, 45].

Gestational diabetes, on the other hand, may negatively impact BF initiation with an effect on mammary development and milk production. Emerging evidence connect different levels of glucose intolerance with poor development of mammary glands during pregnancy [46, 47], delayed onset of lactogenesis [48, 49], and reduced milk production in early and mature lactation [50, 51]. This delayed milk production is thought to be a result of protein tyrosine phosphatase, receptor type F overexpression in the mammary gland caused by decreased insulin sensitivity [50].

Most studies analyzed only the effect of maternal overweight or the effect of gestational diabetes over BF initiation. Matias et al., however, assessed the impact of maternal obesity evaluating GDM women from the Study of Women, Infant Feeding and Type 2 Diabetes After GDM Pregnancy (SWIFT), and found that pre-pregnancy obesity was independently associated with delayed lactogenesis in women with GDM [52]. The authors, however, were not able to identify whether the outcome was caused only by maternal obesity or if obesity and GDM were interacting to produce this effect, since there were no control women without diabetes in the study.

Given all the evidence of worse BF outcome in overweight women, a few intervention studies have been conducted to improve BF initiation rates for this group. Rasmussen et al. conducted two low-intensity interventions with obese women [53]: the first one design to give telephone support to mothers on BF and the second one to provide manual or electric pumps for milk supply. Neither of them were successful in enhancing BF rates. Another study provided intensive peer counseling to overweight/obese mothers, including prenatal visits, daily in-hospital visits, postpartum home visits and phone calls [54]. This intervention also had no impact on BF initiation and on exclusive BF rates at 1, 3, or 6 months postpartum.

Antenatal breast expression is lately being applied, especially to diabetic women, in order to store colostrum and handle neonatal hypoglycemia and as a form of hastening milk production and preventing delayed initiation of BF after delivery [55]. The efficacy and safety of this practice, however, is yet to be proven, with some small studies indicating that this procedure could induce preterm delivery and increase the frequency of NICU admissions [56]. Forster el al. conducted a big randomized control trial and found no harm in advising diabetic women with low risk of complications to express breast milk from 36 weeks’ gestation, however the authors enunciated their concerns with the extrapolation of this results to other high risk groups without further research [57].

Our results were both strengthened and limited by the design of the IVAPSA Study. To our knowledge this is the first study to present women with and without GDM and women with and without overweight/obesity to investigate the interaction of these adverse conditions over BF initiation. Greater statistical power, with the increase in the number of participants in each group, however, would be substantial to carry out more complex statistical analysis. We can not refute the possibility that the aggravation of obesity for women with both overweight and GDM could, in part, be responsible for the increased relative risk for delayed BF in this group. Another strength of this study was the quality of the prospective data collection, especially on BF and maternal anthropometry.

### Conclusion

This study indicates an additive interaction between maternal pre-pregnancy overweight/obesity and gestational diabetes on delayed BF initiation. Intervention studies that aim to improve BF initiation in these target groups are essential given the increasing rates of overweight and diabetes in women of reproductive age worldwide and the benefitial effect of BF for these women and their offspring.

## Supporting information

S1 File MDS_plos: Minimal data set for the analysis presented in this paper. (SAV)

S2 File MDS labels: Labels for the minimal data set. (DOCX)

## Abbreviations

aRR: Adjusted Relative Risk
BF: Breastfeeding
BMI: body mass index
GDM: gestational diabetes
IVAPSA: Impact of Perinatal Different Intrauterine Environments on Child Growth and Development in the First Six Months of Life Study
SD: Standard Deviation
SPSS: Statistical Package for the Social Sciences
WHO: World Health Organization
