# Supplementary material for: Maternal pre-pregnancy overweight/obesity and gestational diabetes interaction on delayed breastfeeding initiation
Source: PLoS One. 2018 Jun 18;13(6):e0194879. doi: 10.1371/journal.pone.0194879 (PMC6005508; doi:10.1371/journal.pone.0194879)
Supplement: S2 File — (DOCX) [file pone.0194879.s002.docx]

Minimal Data Set Labels – PLOS ONE Pinheiro, T.V.

IDENTIF – IVAPSA study identification number

DM – gestational diabetes diagnosis (0, yes; 1, no)

IDADEMAE – participant’s age (years)

PARID – participant’s parity (number of previous live births)

PARIDCAT – categorized parity (0, none; 1, one or more)

ESCOL – participant’s schooling (years)

ESCOLCAT – categorized schooling (0, less than eight years; 1, eight or more years)

TIPARTO – type of delivery (0, cesarean; 1, vaginal)

HR1MAM – timing of breastfeed initiation after birth (minutes)

MAMD1CAT – breastfeeding initiation categorized (0, more than 24 hours after delivery; 1 less or equal to 24 hours after delivery)

PESOPG – pre-pregnancy weight (kg)

ALTURAMD – height (m)

IMCPG – pre-pregnancy BMI (kg/m^2^)

IMCPGCAT – categorized pre-pregnancy BMI (Normal weight: 18.5–24.9 kg/m2; Overweight: 25.0–29.9 kg/m2)
